# Comparative Analysis of the Equivital EQ02 Lifemonitor with Holter Ambulatory ECG Device for Continuous Measurement of ECG, Heart Rate, and Heart Rate Variability: A Validation Study for Precision and Accuracy

**DOI:** 10.3389/fphys.2016.00391

**Published:** 2016-09-21

**Authors:** Abimbola A. Akintola, Vera van de Pol, Daniel Bimmel, Arie C. Maan, Diana van Heemst

**Affiliations:** ^1^Department of Internal Medicine, Section Gerontology and Geriatrics, Leiden University Medical CenterLeiden, Netherlands; ^2^Department of Cardiology, Leiden University Medical CenterLeiden, Netherlands

**Keywords:** ECG, Equivital-EQ02, Holter electrocardiogram, remote monitoring, validation study, heart rate, heart rate variability (HRV), ECG artifact processing

## Abstract

**Background:** The Equivital (EQ02) is a multi-parameter telemetric device offering both real-time and/or retrospective, synchronized monitoring of ECG, HR, and HRV, respiration, activity, and temperature. Unlike the Holter, which is the gold standard for continuous ECG measurement, EQO2 continuously monitors ECG via electrodes interwoven in the textile of a wearable belt.

**Objective:** To compare EQ02 with the Holter for continuous home measurement of ECG, heart rate (HR), and heart rate variability (HRV).

**Methods:** Eighteen healthy participants wore, simultaneously for 24 h, the Holter and EQ02 monitors. Per participant, averaged HR, and HRV per 5 min from the two devices were compared using Pearson correlation, paired *T*-test, and Bland-Altman analyses. Accuracy and precision metrics included mean absolute relative difference (MARD).

**Results:** Artifact content of EQ02 data varied widely between (range 1.93–56.45%) and within (range 0.75–9.61%) participants. Comparing the EQ02 to the Holter, the Pearson correlations were respectively 0.724, 0.955, and 0.997 for datasets containing all data and data with < 50 or < 20% artifacts respectively. For datasets containing respectively all data, data with < 50, or < 20% artifacts, bias estimated by Bland-Altman analysis was −2.8, −1.0, and −0.8 beats per minute and 24 h MARD was 7.08, 3.01, and 1.5. After selecting a 3-h stretch of data containing 1.15% artifacts, Pearson correlation was 0.786 for HRV measured as standard deviation of NN intervals (SDNN).

**Conclusions:** Although the EQ02 can accurately measure ECG and HRV, its accuracy and precision is highly dependent on artifact content. This is a limitation for clinical use in individual patients. However, the advantages of the EQ02 (ability to simultaneously monitor several physiologic parameters) may outweigh its disadvantages (higher artifact load) for research purposes and/ or for home monitoring in larger groups of study participants. Further studies can be aimed at minimizing the artifacts.

## Introduction

With cardiovascular diseases still representing a leading cause of death globally, continuous electrocardiography (ECG) measurement is becoming increasingly important. Continuous ECG measurements yields valuable information on heart rate (HR) and its variability (HRV) that can be measured at a beat-to-beat level. Their direct clinical importance has been demonstrated in numerous studies. HR is a major risk factor for morbidity and mortality in cardiovascular diseases (Diaz et al., 2005; Hjalmarson, 2007). Even in apparently healthy individuals, HR has predictive value for sudden cardiac death (Mølgaard et al., 1991). Furthermore, control of HR has become the focus of drug development for cardiovascular diseases (Routledge et al., 2002). In addition to HR, continuous measurement of heart rate variability (HRV) serves as an index of cardiac sympathetic and parasympathetic activity (Thayer et al., 2010). HR and HRV associate with cardiac (Mølgaard et al., 1991), physiological (Tsuji et al., 1994), psychological (Friedman and Thayer, 1998; Dishman et al., 2000), and sleep-related disorders (Stein and Pu, 2012); and are being used as prognostic indicators for cardiac- and non-cardiac diseases, such as idiopathic dilated myopathy (Fauchier et al., 1997), myocardial infarction (La Rovere et al., 1998), renal failure (Oikawa et al., 2009), end stage renal disease (Hayano et al., 1999), and cancer (Guo et al., 2015).

ECG signals can be obtained from varying sources, such as Holter monitoring, bedside monitoring of vital parameters, systems for surface ECG, ergometric stress tests, and systems for telemetry (Gulizia et al., 2006). Of these, the Holter monitor (Holter) is the gold standard for continuous ECG measurement. The Holter records ECG signals via electrodes attached to the chest. However, over the years different innovative devices that are able to comfortably monitor ECG simultaneously with other physiological parameters for a prolonged period of time in freely moving subjects have become available. The Equivital EQ02 Lifemonitor (EQ02,) is a convenient and safe wireless ambulatory device that continuously measures ECG, HR, and HRV via a chest-worn sensor belt embedding textile-based electrodes. In addition to cardiac parameters, EQ02 also measures breathing rate, body position, and movement (accelerometry), and skin and core body temperature, all synchronized- and time- stamped to provide contextual significance for possible diagnostic, therapeutic, or research purposes. Although EQ02 has been used in several studies, e.g., for ambulatory monitoring of pilots, athletes, and military personnel, both under physiological and extreme environmental conditions (Karlen et al., 2010; Bizzini et al., 2013; Liu et al., 2013; Tharion et al., 2013), EQ02 has not yet been validated against the gold standard for measurement of cardiac parameters.

Here for the first time, we compared the accuracy of EQ02 and Holter for continuous ECG, HR, and HRV monitoring. The EQ02 and Holter were worn simultaneously for 24 h in home setting by an heterogeneous group of healthy male and female volunteers. Results were analyzed in point accuracy (including absolute and relative differences), monitor reliability, and precision metrics for both devices.

## Methods

### Ethics statement

This study was approved by the institutional review board of Leiden University Medical Center (LUMC) under protocol P11.116. All study participants gave written informed consent.

### Study participants

The present study was embedded in the Switchbox Study, which was a sub-study of the Leiden Longevity Study (LLS). The LLS was originally designed to investigate genetic and phenotypic biomarkers associated with human longevity. A more detailed description of the study design and recruitment strategy for the Switchbox study (Jansen et al., 2015) and the LLS (Schoenmaker et al., 2006) has been described elsewhere. The present study population consisted of 18 healthy adult male and female volunteers from the local population. The only exclusion criterion was presence of obvious chest deformity, which would impair lifemonitor belt fitting.

Apart from the 18 subjects, artifact percentage was determined in all raw Holter recordings that were collected in the department of Cardiology of the LUMC in 2014. In total, artifact data from ECG recordings of 4143 persons were used. Apart from the percentage of artifacts contained in the recordings, no other data from these individuals were used. Furthermore, similar raw artifact data were extracted from EQ02 recordings from 200 switchbox participants.

### Experimental protocol

After body mass index and waist: hip ratio were measured, participants wore, simultaneously, the EQ02 monitor, a Holter, and a Fitbit one™. These were turned on approximately concurrently. Participants undertook their usual daily activities, except swimming. They additionally kept a detailed diary of the type and timing of all their activities.

### Study devices

#### EQ02 monitoring

The EQ02 (Equivital EQ02, Hidalgo, UK) continuously measured ECG on two channels via three electrodes (Table 1). The EQ02 monitoring system consisted of a LM 1000 Lifemonitor sensor electronic module (SEM), Lifemonitor belts of varying sizes, a SEM lead and charging dock, a blue tooth USB dongle for laptop/ PC, and an Equivital Manager to configure SEMs and to download and export data. For this study, SEMs were configured in clinical mode, and data reported retrospectively at local time. Bluetooth connectivity was disabled and data transmission was at partial disclosure.

**Table 1:** **Technical characteristics of the EQ02 and Holter monitors**.

	**Holter**	**Equivital (EQ02) lifemonitor**
Acceptance	Gold standard	Relatively new device
Parameters measured	ECG only	ECG, breathing rate, tri-axial accelerometry, skin temperature, core body temperature, all fully synchronized.
Data presentation	Retrospective	Real-time (live) and retrospective (date- and time- stamped).
Recording modes	Ambulatory	Ambulatory and Clinical
Recording time	Usually 24–48 h	Fully charged battery lasts 24–48 h^*^. The internal memory of the recorder stores up to 50 days of data
Channels	3	2
Electrodes	7	3
Type of electrodes	Stick-on	Textile electrodes
Skin preparation	Necessary (Removal of non-conductive skin layer)	None
Convenience	Can be cumbersome due to multiple lead wires, sensor pads, clips and/or re-enforcing tapes, carry-case	Easy to wear belt
Analysis software	MARS	Vivosense
Data quality	Gold standard	High quality P-wave and QRST detection. Accurate and precise ECG, HR and HRV measurement when artifact content of its recording is < 20%

* For this study, the monitors were charged after 12 h of use.

An appropriately sized lifemonitor belt held the SEM onto the subject's body. Its textile-based electrodes were moistened with water before making contact with the participant's skin. SEMs were charged for approximately 1 h after 12 h of recording. Upon study completion, SEM data was uploaded onto the Equivital manager; from where date- and time- stamped ECG, inter-beat interval, and summary data of vital signs were extracted and exported.

#### Holter ECG monitoring

**Figure 1:**
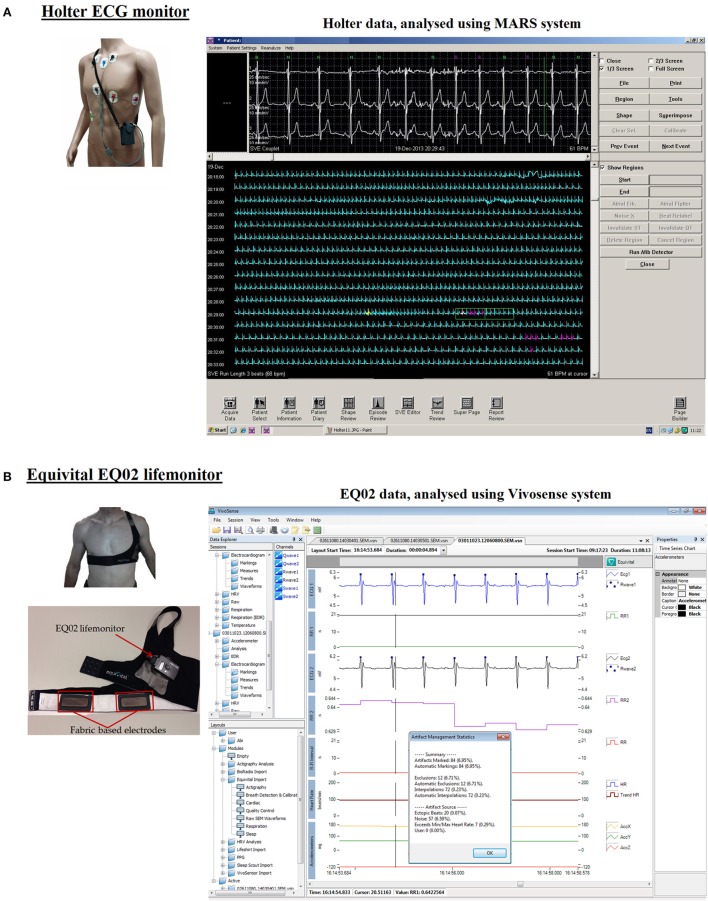
**Analysis software for Holter and EQ02 data management. (A)** Shows the Holter monitor including its electrodes, lead wires, and its analytical software. Annotated tracings from the Holter can be seen on the MARS software. **(B)** Shows the EQ02 unit, consisting of the lifemonitor belt on which are three textile-based electrodes. ECG tracings are visualized on the cardiac layout of Vivosense software.

The Holter (SEER MC Holter monitor, GE Healthcare, USA) measured ECG on three channels (Table 1). The Holter consisted of seven electrodes; color-coded lead wires and a battery operated, digital ECG recorder. Before placement of electrodes, participants' skin were prepared with alcohol and 3M red dot 2236 trace prep (3M Healthcare, Canada) to remove non-conductive skin layer and reduce skin impedance and eventual artifacts. Color-coded leads were clipped on to 3M electrodes (type 2271, 3M Healthcare, Canada) and placed as shown in Figure 1.

#### Fitbit one™ wireless activity and sleep tracker

The Fitbit one™ (Fitbit, San Franscisco, USA) was worn on the waist (belt) during the day for tracking activity (step-counts) and on the sleep wrist-band at night for tracking sleep length and number and durations of awakenings. Upon study completion, data was downloaded via Fitbit dashboard. Sleep efficiency was extracted as a composite of time to fall asleep, number of awakenings, and restless periods, total time in bed and the actual sleep time (Di Rienzo et al., 2013).

### Data management

While data extracted from EQ02 were automatically time- and date stamped (date, time in hr., min., sec. & ms.), Holter data were not. Data from both devices were synchronized based on the Equivital time stamp, by selecting aberrations (non-sinus beats) in two consecutive heart beats from the Holter that corresponded to those from EQ02, mostly around the start of the recording. 5-min trends (averages) of HR, RR, and HRV parameters from synchronized data were then extracted for analysis.

#### Data management: holter monitor

Holter ECG data were analyzed using MARS ambulatory Holter ECG analysis system (GE Healthcare, Milwaukee, WI, USA). After extraction, an exportable file was visible on MARS, which contained the annotations “N” (normal sinus-rhythm), “V” (ventricular-beat), “S” (supraventricular-beat), or “X” (artifact; Figure 1A). The software recognized and grouped QRS complexes on similarity. This process was manually checked and corrected when the recognition of QRS-location was faulty or the sinus/non-sinus labeling was wrong.

#### EQ02 data management: vivosense

Raw ECG data from EQ02 was analyzed using Vivosense modular physiological monitoring and analysis platform (Vivonoetics, San Diego, USA). EQ02 data were visualized with *Cardiac* layout (Figure 1B), for inspecting each ECG channel and derived R-wave markings, and artifact identification. This layout also contained accelerometer data channels for contextual interpretation.

The EQ02 unit provided two leads of ECG measurements that shared a common reference. These were denoted as SEM_ecg1 (primary raw ECG signal) and SEM_ecg2 (secondary raw ECG signal) respectively. Vivosense processed and performed QRS detection on both channels to generate two sets of R-wave markings. We chose SEM_ecg1, which was then scaled, and filtered by Vivosense, as primary source ECG for derivation of RR, HR, and HRV parameters.

Artifacts in the ECG signals were identified and annotated. Artifacts were defined as (i) distorted signals and/ or (ii) segments of signals in which the different waves of the ECG complex could not be clearly identified. Vivosense offers an algorithm that automatically marks and calculates artifact percentage (Figure 1B), and no-, low-, medium-, and high-artifact cleaning/ noise reduction options. The automatic artifact-marking algorithm takes into account the minimum and maximum allowable heart rates, presence of ectopic beats, maximal interpolation length, and signal noise. After removal of charging times, Vivosense automatic cleaning of the data in this study was performed by selecting the timeframe to be cleaned, setting the sensitivity level of the automatic cleaning algorithm at medium noise filtering, and setting the minimal and maximal allowable HR limits to 30 and 220 beats per minutes respectively.

In addition, complete manual cleaning of EQ02 data was done for one participant, involving the time-intensive process of relocating incorrectly automatically recognized QRS-complexes to correct locations, and manually identifying and excluding artifacts.

Furthermore, Vivosense software calculated and displayed eight HRV indices, namely, average of NN-intervals (ANN), standard deviation of NN-intervals (SDNN), standard deviation of 5-min averages of NN-intervals (SDANN), standard deviation of successive differences of NN-intervals (SDSD), square root of the mean squared differences of successive intervals (RMSSD), mean of the standard deviation of 5-min NN-intervals (SDNNi), number of adjacent NN-intervals with a difference less than 50 ms (NN50), and ratio of NN50 to total number of NN-intervals (pNN50).

### Accuracy, precision, and reliability metrics

The point accuracy of EQ02 was measured in terms of the relative difference (RD) and absolute relative difference (ARD) of HR measurements to assess respectively the bias (relative to the Holter) and the average error. The RD was calculated using the formula [(EQ02 HR—Holter HR)/Holter HR]. The ARD was calculated using the formula [|EQ02 HR—Holter HR|/Holter HR]. We additionally determined the mean absolute relative difference (MARD) of all paired points. The mean and standard deviation (SD) of MARDs from all 18 participants were computed for each synchronized 24 h HR measurement, to assess respectively the accuracy and precision of EQ02 HR measurements.

### Statistical analysis

Of the original 5182 paired data points of 5-min HR averages from 18 participants, 4736 (91.4%) remained after exclusion of charging times. From these, three datasets were made containing: (1) raw data (all 4736 data points) (2) filtered data containing < 50% artifacts [4059 (85.5%) data points] (3) filtered data containing < 20% artifacts [3677 (77.6%) data points].

To analyze the strength of the linear relationship and agreement between both devices, synchronized data from both devices were analyzed with Pearson correlation analysis, and Bland-Altman plots for all three datasets. To explore possible determinants of artifacts, we stratified data based on sex, day, and night, tertiles of waist: hip ratios, BMI, and tertiles of activity. The artifact distribution in strata was compared using Chi-square (*X*^2^) test. The association between BMI and artifact load was assessed using linear regression.

The per-participant estimates of HR and HRV derived from EQ02 and Holter monitors were compared with paired *t*-tests. For all paired points, RD, ARD, and MARD were determined using aforementioned formulae.

Graphs were drawn using GraphPad Prism version 5 (GraphPad, San Diego, CA). All statistical analyses were performed using SPSS v.20 (SPPS Inc., Chicago, U.S.A.). Two-sided *p*-values below 0.05 were considered statistically significant.

## Results

### Heart rate

Characteristics of study participants are summarized in Table 2 and described in detail per-person in Supplementary Table 1. The mean age of the participants was 27.6 years (range 19–57 years); 10 (55%) were males. The activity pattern of the participants was variable, ranging from 5017 to 14,265 steps taken in 24 h. Medical history showed that none of the participants had persistent chest pain, tiredness, dyspnea, lightheadedness, palpitations, cardiovascular diseases, hypertension, endocrine, or other diseases. Two participants had hypothyroidism, for which they used levothyroxine (data not shown).

**Table 2:** **Subject characteristics**.

**Demographics**	***N* = 18**
Male n (%)	10 (55)
Age, years	27.6 (9.4)
Weight, kg	72 (10.2)
BMI, kg/m2	22.5 (2.4)
Waist: hip ratio	0.81 (0.1)
Sleep (mins)	580.2 (156)
Step counts (total in 24 h)	9635 (2916)
% Artifacts in raw EQ02 data^*^	19.0 (14.7)

Data represent mean with standard deviation unless stated otherwise.

* Excluding charging times.

Different components of the cardiac cycle, including p-waves were clearly identifiable in the ECG tracings from both the EQ02 and Holter (Figure 1).

#### Artifact management

As shown in Table 2, the average artifact percentage of the 24 h EQ02 data was 19% (SD 14.7). However, marked differences existed in data quality (Supplementary Table 1) between participants (range 1.93–56.45%) and within participants (range 0.75–99.61%). Individual 24 h graphs of Holter and EQ02 heart rate measurements of each of the 18 participants are shown in Supplementary Figure 1.

**Figure 2:**
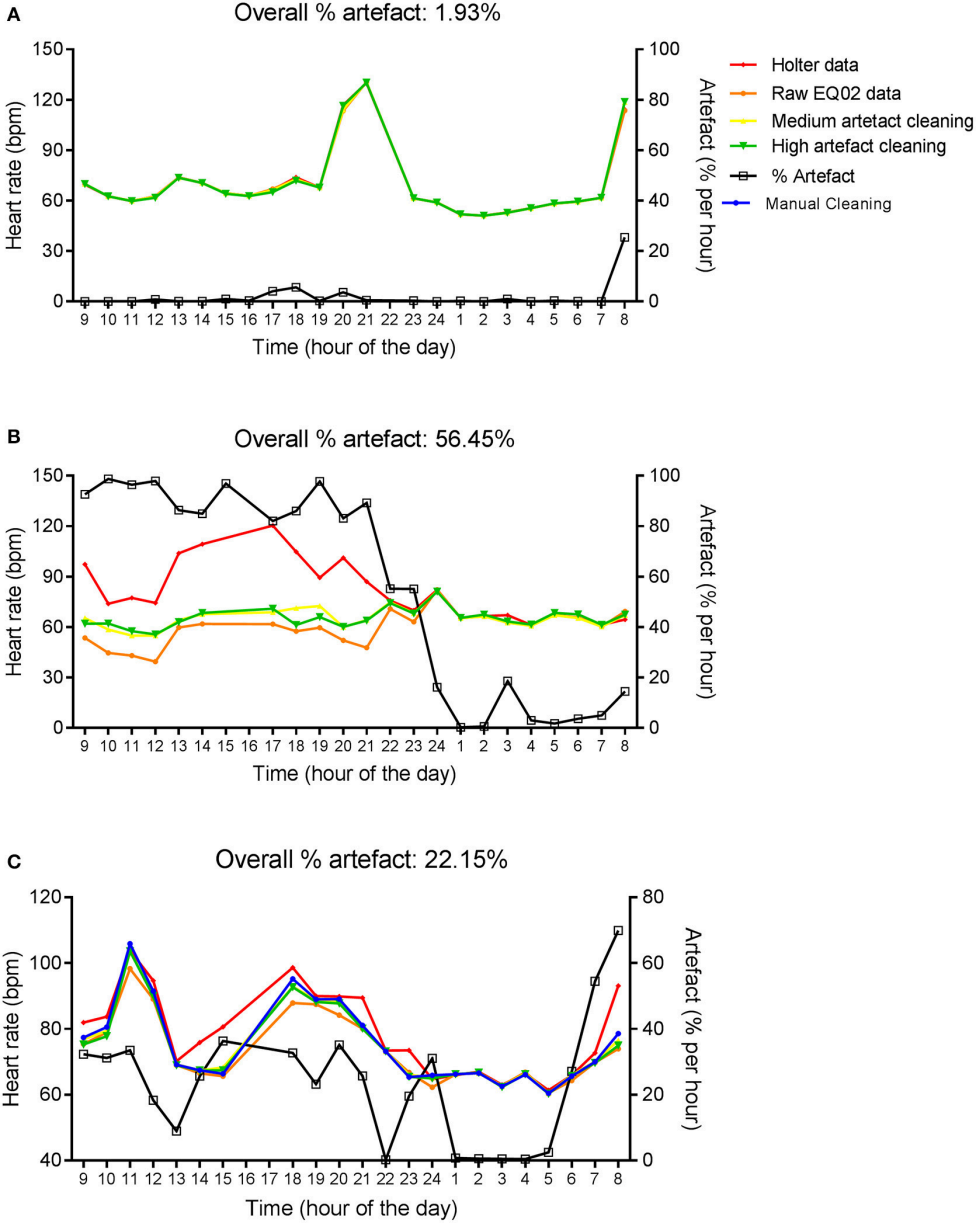
**Twenty four hour HR measurements by EQ02 and Holter**. Hourly averages of HR measured simultaneously using the EQ02 and Holter monitors for three participants **(A–C)**. Raw EQ02 and automatically cleaned EQ02 data using the medium and high artifact sensitivity options of the Vivosense software are displayed. Artifact percentages over 24 h (above each graph) and per hour (right y-axis) are presented.

Figure 2 shows hourly averages of HR from the Holter, and EQ02 before cleaning, and after medium and high sensitivity cleaning for three representative participants with average artifact percentages over 24 h of 1.93, 56.45, and 22.15% respectively. Artifact percentages were variable throughout the day, but higher just around charging times, and lowest at night. At lower artifact percentages, there was good concordance between the EQ02 and Holter HR. In contrast, at higher artifacts percentages, there was discordance between the EQ02 and Holter HR values that persisted after applying the Vivosense automatic cleaning methods.

In addition, 24 h EQ02 data for the participant in Figure 2C was cleaned manually, which took 24 h. Manual cleaning did not eliminate the discordance between Holter and EQ02 data at high artifacts percentages.

#### EQ02 sensor performance

**Figure 3:**
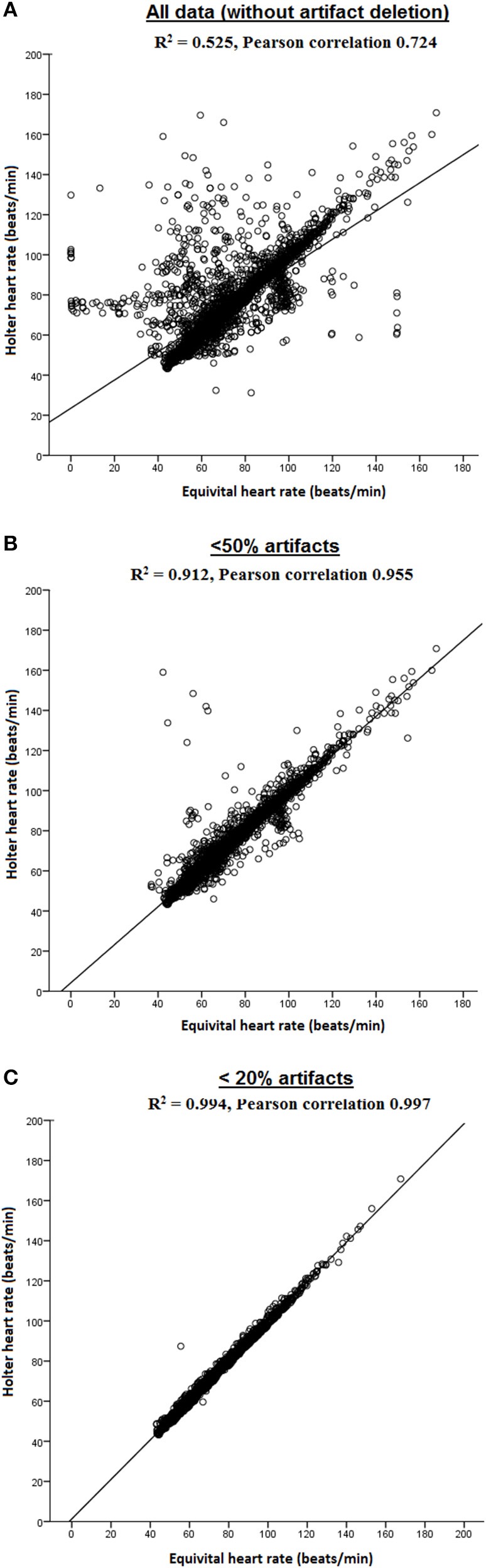
**Pearson correlations of HR measured by EQ02 and Holter**. Both *R*^2^ and Pearson correlation coefficients are shown for **(A)** all data, and filtered data containing **(B)** < 50% artifacts and **(C)** < 20% artifacts.

Figure 3 displays the Pearson correlation coefficients of HR measured using EQ02 and Holter for the three datasets sorted on artifact percentage. Pearson correlations were 0.724 for all data, and 0.955 and 0.997 for the datasets containing < 50 and < 20% artifacts respectively.

#### EQ02 point accuracy, mean accuracy, and precision metrics

**Figure 4:**
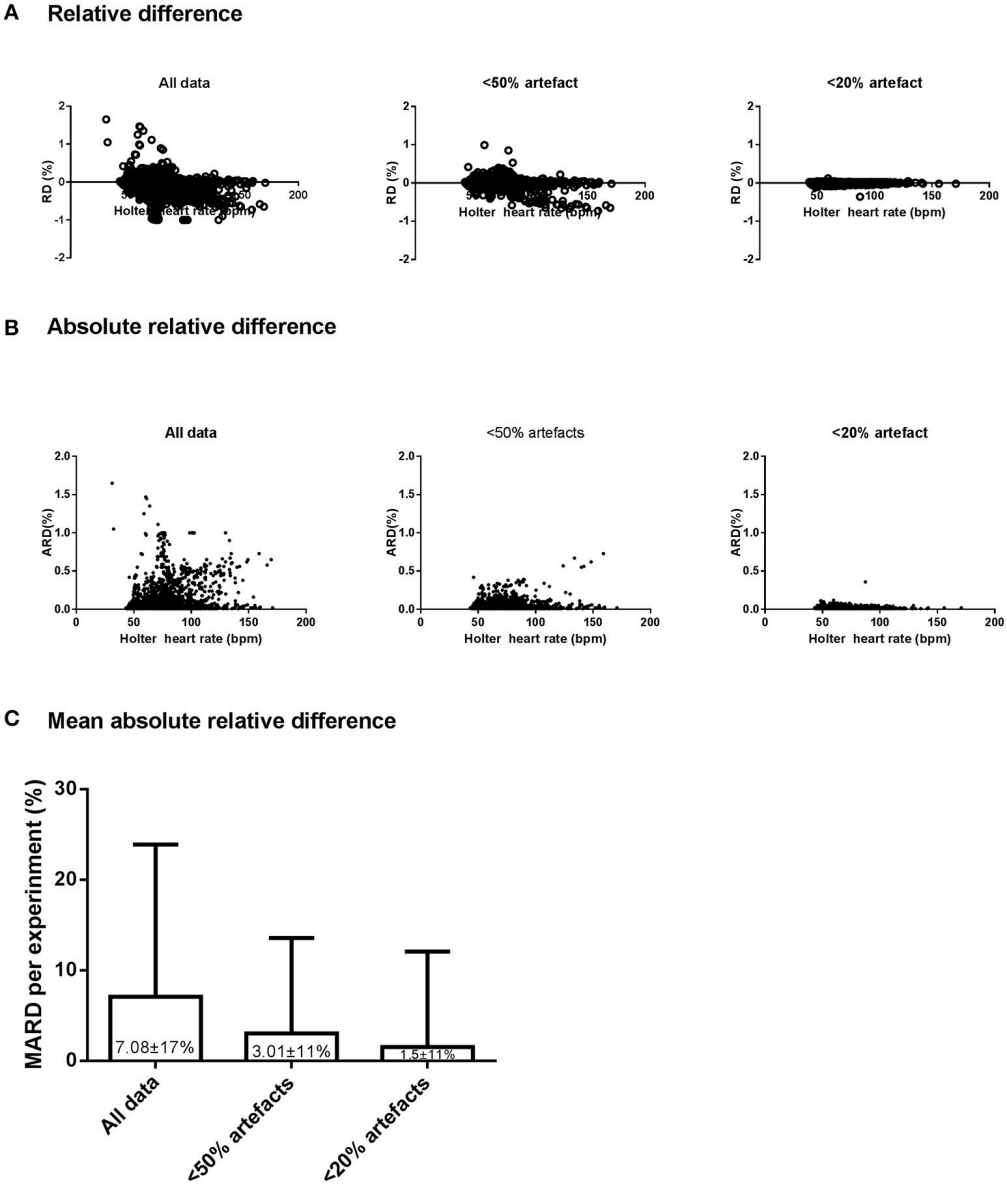
**Accuracy, precision, and reliability of the EQ02 HR measurements**. The distribution, as a function of Holter values, of the relative difference (RD, **A**) and absolute RD **(B)** between each EQ02 HR measurement and its corresponding Holter HR value, for all data, and filtered data containing < 50% and < 20% artifacts respectively. **(C)**: MARD between EQ02- and Holter- derived HR values.

The point accuracy for EQ02 across the three datasets of varying artifact percentages are shown as RD and ARD distributions in Figures 4A,B. For all data, 2246 of 4542 (49%) paired RD points had negative RD values. From the datasets containing < 50% artifacts and < 20% artifacts, respectively, 1802 of the 3882 (46%) and 1359 of the 3118 (43.6%) paired RD points had negative RD values. From the distribution of RD and ARD values shown in Figure 4, the distribution of the underestimation extended over a broader range of HR values at higher artifact percentages.

As an indicator of mean accuracy and precision of the EQ02, the MARD in EQ02 HR data relative to Holter HR over 24 h are presented for the three data sets in Figure 4C. The 24 h MARD was 7.08 ± 17% for all data, 3.01 ± 10.55% for data containing < 50% artifacts and 1.5 ± 10.51% for data containing < 20% artifacts. As depicted by the SD of the MARD, while the precision did not markedly differ between < 20% (SD = 10.51) or < 50% artifacts (SD = 10.55), precision decreased at >50% artifacts (SD = 17).

#### Agreement between devices

**Figure 5:**
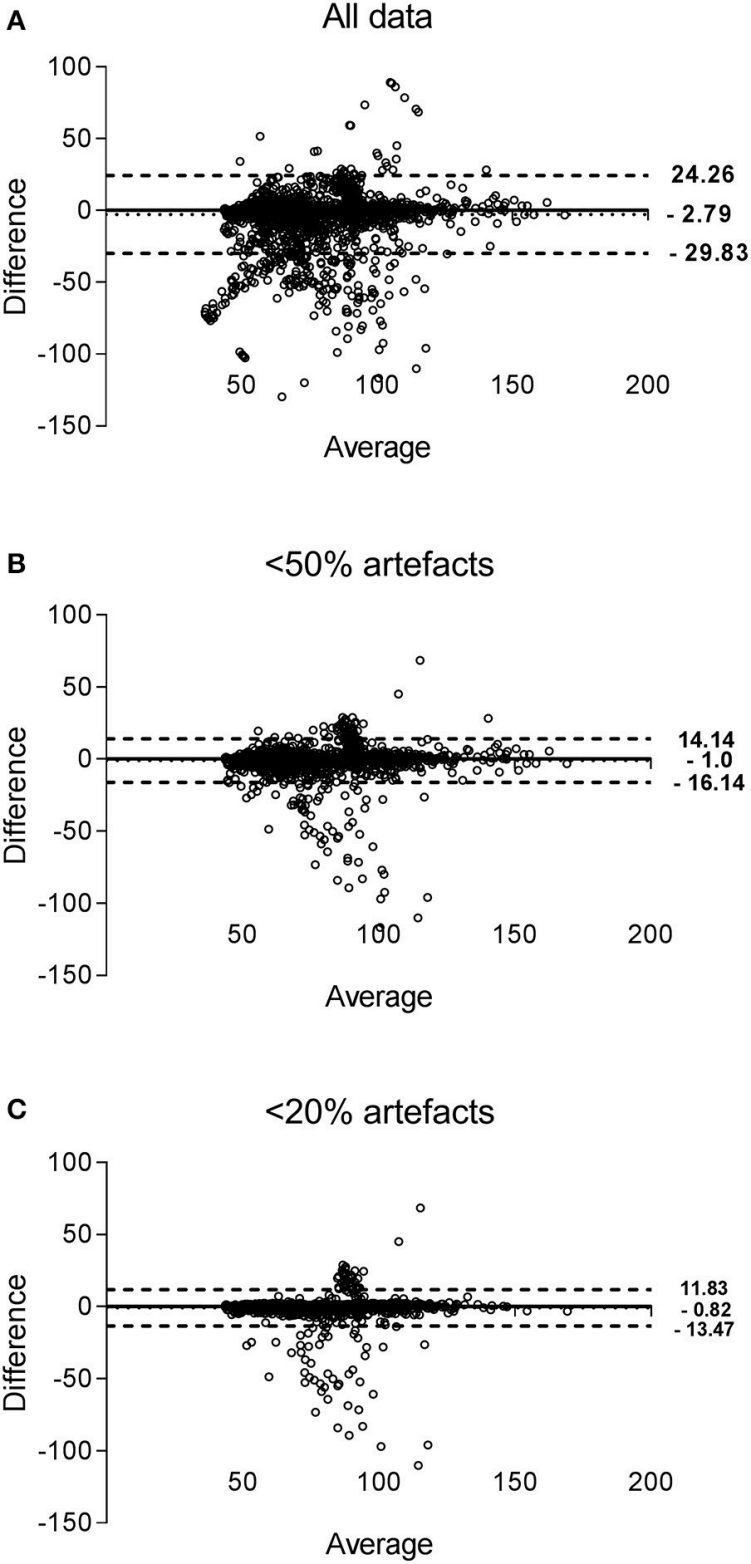
**Bland-Altman plots of HR measured by EQ02 and Holter**. Each dot represent paired (EQ02-Holter) HR values derived from all participants. The bias of the measurements (represented as solid lines) and the ± 1.96 SD (dotted lines) are presented for the measurements obtained for all data **(A)**, filtered data containing < 50% **(B)** artifacts and < 20% artifacts **(C)**.

Bland-Altman plots of paired HR values from both devices are presented in Figure 5, for the three datasets. Compared to the Holter, HR was on average lower when derived from EQ02, with respectively a mean (95% CI) difference of −2.8 (−29.8 to 24.3) beats per minute (bpm) for all data, −1.0 (−16.1 to 14.14) bpm for data < 50% artifact and −0.8 (−13.5 to 11.8) bpm for data < 20% artifacts.

#### Evaluation of artifacts

**Figure 6:**
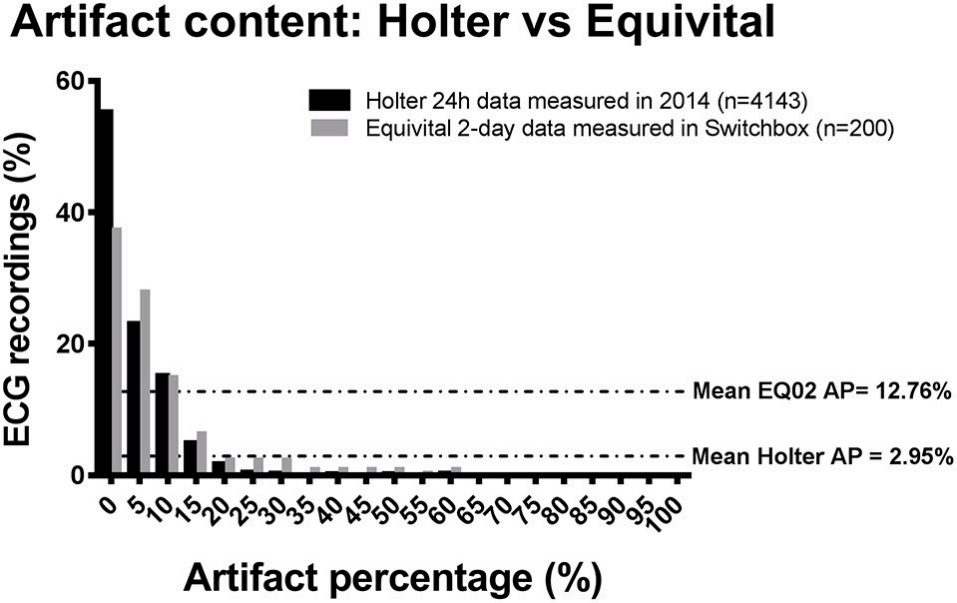
**Comparison of artifact content of raw Holter data to raw EQ02 data**. Bar chart showing content and distribution of artifact in raw (uncleaned) 24 h Holter ECG recordings from 4143 subjects with raw EQ02 data from 200 subjects. The average artifact percentage from the holter recordings was 2.95%, whereas the average artifact percentage from that 200 EQ02 recordings was 12.76%. AP: artifact percentage.

In order to evaluate the artifact burden of the EQ02, we compared the artifact content of 24 h Holter ECG recordings from 4143 subjects with raw EQ02 data from 200 subjects. The average artifact percentage from the 4143 holter recordings was 2.95%, whereas the average artifact percentage from the 200 EQ02 recordings was 12.76%. Thus, the mean difference in artifact percentage in raw EQ02 compared to raw holter ECG was 10%. For the Holter, 77.9% of the 4143 raw Holter ECG recordings had artifact percentage of ≤ 5%, whereas 65.5% of the 200 raw EQ02 ECG recordings had artifact percentage of ≤ 5%. The distribution of the artifacts is presented in Figure 6.

Next, we evaluated possible sources of artifacts for the EQ02 recordings, including sex, waist: hip ratio, daytime vs. nighttime (during which participants were asleep), activity (step counts) and BMI. The mean (SD) artifact percentages of male participants [19.4% (11.4)] was not significantly different (*P* = 0.348) from that of females [15.9 (17.9)]. When participants were divided into tertiles based on waist: hip ratio (w:h), tertile 1 (w:h range 0.71–0.76) had mean (SD) artifact percentage of 7.5 (3.5); tertile 2 (w:h range 0.77–0.83) had mean (SD) artifact percentage of 16.4 (6.0) while tertile 3 (w:h range 0.87–0.92) had mean (SD) artifact percentage of 29.7 (18.5) (*P* = 0.014).

During the day, 62.9% of the data contained < 20% artifacts, 17.15% contained 20–50% artifacts and 19.9% had 50–100% artifacts. In contrast, during the night, 82.7% of the data contained < 20% artifacts, 12.4% contained 20–50% artifacts whereas 4.9% had 50–100% artifacts. Thus, there was considerably more artifacts during daytime compared to nighttime (*p* < 0.001).

Furthermore, we also evaluated if activity of the participants, as measured using number of step counts taken during the study, had a bearing on artifacts percentage. Participants were divided into tertiles based on step counts, representing low activity (tertile 1, with 5017–8228 steps), medium activity (tertile 2 with 8229–11,439 steps), and high activity (tertile 3 with 11,440–14,265 steps) respectively. For tertile 1 (least active), 73.3% of the data contained < 20% artifacts, 11.8% contained 20–50% artifacts, and 14.9% of the data from the least active people contained 50–100% artifacts. For tertile 2 (medium active), 66.2% of the data contained < 20% artifacts, 14.7% contained 20–50% artifacts, and 19.0% of the data contained 50–100% artifacts. Thus, there were comparatively more artifacts in tertile 2 compared to tertile 1 (*p* < 0.001). Similar significant result was obtained for comparison of tertile 3 (most active) to tertile 1 (*p* < 0.001).

Finally, we assessed the association between BMI and artifact percentage. No significant association was found between BMI and artifact content of the EQ02 ECG recordings (*P* = 0.256), data not shown.

### Heart rate variability

**Figure 7:**
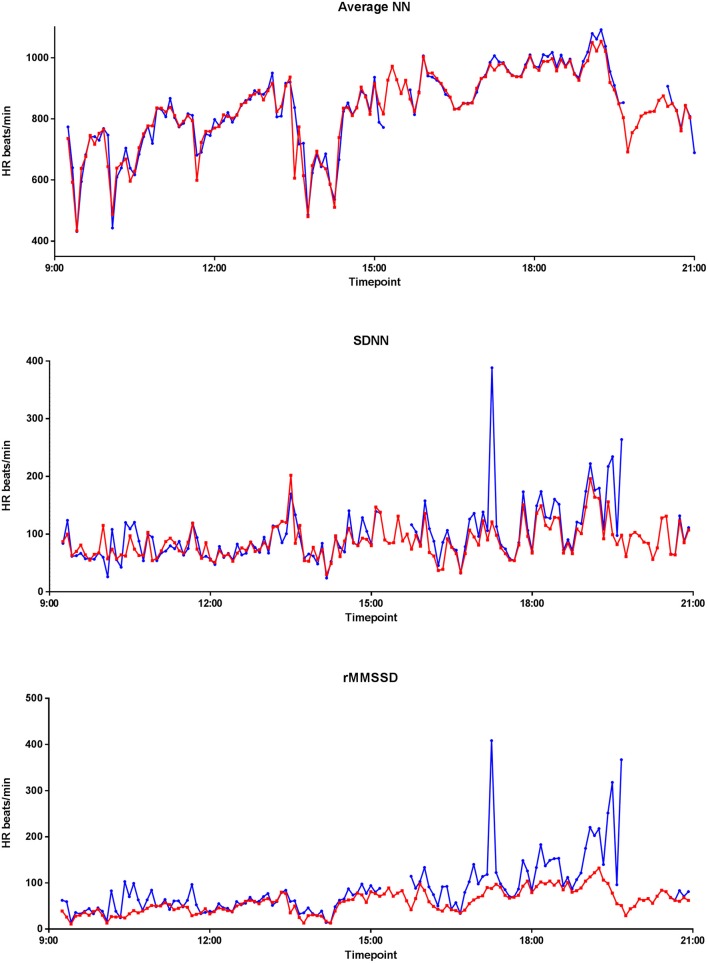
**Twenty four hour EQ02 and Holter HRV profile**. Average NN, SDNN, and rMMSD of a participant over 24 h, as recorded by the Holter (red) and EQ02 (blue).

**Figure 8:**
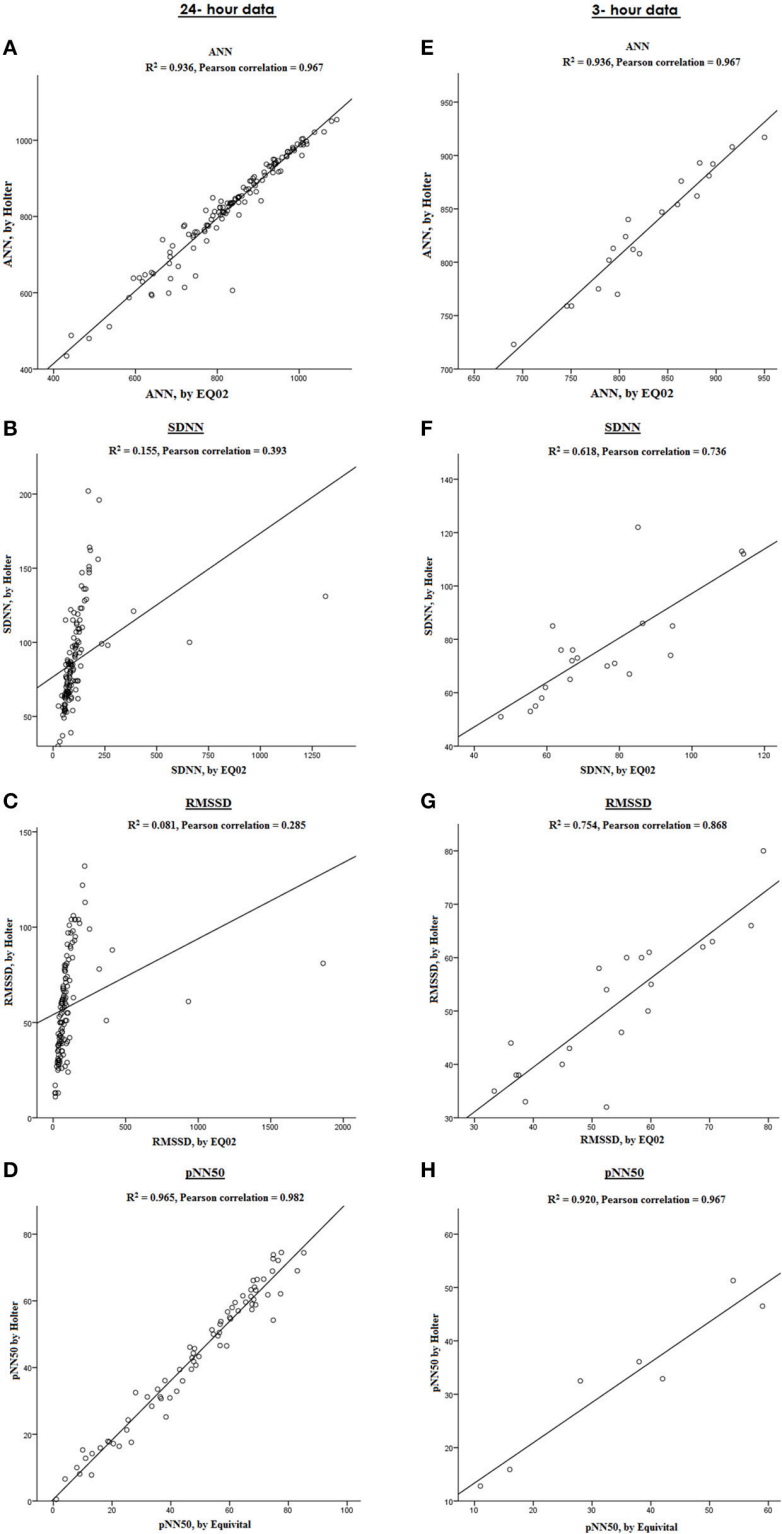
**Comparison between HRV parameters measured by EQ02 and Holter**. Correlation between ANN, SDNN, RMSSD, and pNN50 in a participant from both devices over 24 h **(A–D)** and in a sub-selection of 3-h data with artifact percentage of 1.15% **(E**–**H)**.

Figure 7 graphically displays 10-min averages of different HRV parameters for one participant (serial number 11, with overall average artifact percentage of 5.23%). Comparing HRV parameters from the two devices, the Pearson correlations were 0.967 for ANN, 0.393 for SDNN, 0.285 for rMSSD, 0.680 for SDANN, and 0.982 for pNN50 for the 24-h data. However, after selecting a 3-h stretch of data containing minimal artifacts (1.15% artifacts), the Pearson correlation for ANN remained the same. Except for pNN50 with Pearson correlation of 0.967 for 3-h data, the Pearson correlation coefficients of the other HRV parameters improved to 0.786 for SDNN, 0.868 for rMSSD, and 0.991 for SDANN for the 3 h data with minimal artifacts (Figure 8).

## Discussion

The EQ02 is a wireless device which can be used for monitoring multiple parameters, either real-time/live or offline/retrospectively. This is the first study to investigate the accuracy of EQ02 for continuous ECG measurement by comparing EQ02 with the gold standard (Holter). The major findings of this study are: (1) EQ02 is a convenient device for continuous measurement of ECG and its derivatives; (2) marked differences were observed in data quality between and within participants; (3) at lower artifacts percentages, HR, and HRV measurements from EQ02 and Holter measurements were highly correlated; (4) artifact percentages were lower during nighttime, when waist: hip ratio was lower, and at lower activity/ movement levels, as measured by step counts taken during the study.

EQ02 is relatively easy to wear in a home setting during habitual activities due to the design of the belt system with textile electrodes (the absence of wires). The use of wireless fabric electrodes in the wearable belt provides comfort that makes the system suitable for prolonged and/ or frequent recordings (Yu-Hong et al., 2012). However, this also imposes a limitation because textile electrodes are more prone to motion artifacts which interfere with R-wave detection (Nangalia et al., 2010). Since textile—based electrodes do not have adhesives or clips, the instability and misplacement of the electrodes can be a possible source of the relatively higher artifact percentages that we observed for the ECG recordings from the EQ02 monitor. This validation study found marked differences in EQ02 data quality between and within participants, as determined by the percentage of artifacts. We found more artifacts just before EQ02 charging times. For this study, the EQ02 was charged for 1 h for every 12 h of use. Removal and replacement of the monitor around charging times is a possible reason for the increased artifacts around charging times. Known sources of artifacts for cardiac telemetric devices include electrode movement with respect to the skin interface (disrupting electrochemical equilibrium); muscle contraction resulting in unwanted electromyographic contamination that may share the desired signal frequency band; vocalizations; temperature changes; sensor-cross-talk; optical path length changes and electromagnetic induction (Nangalia et al., 2010; El-Sherif and Turitto, 2015). In literature, artifact load of cardiac telemetric devices have also been attributed to body movement, temporary impairment of skin electrode contact, loose electrode connections, broken leads, skeletal myopotentials, and ambient noise (Brage et al., 2005). In line, we also found that higher artifacts were found at higher activity levels, since this involves increased body movement.

Previous studies have shown that when comparing ambulatory electrocardiographic monitoring (AEM) devices, artifacts were more common in telemetric recordings (Brage et al., 2005). When comparing artifact content of the raw EQ02 data to raw Holter data, we found that the difference was 10%. This is higher than the difference of 4.8% higher artifacts that have been reported for other AEMs (Brage et al., 2005), although the comparisons of artifact percentage in previous studies are based on rate of misdetection of arrhythmias. The artifact comparison in the present study was based on artifact content of raw ECG signals from both devices.

The Vivosense software offers three artifact-cleaning options. These are based on the assumption that physiological changes will occur slowly over several beats, whilst artifacts may result in sudden or more rapid changes or non-physiological variability in inter-beat interval timing. Vivosense therefore re-samples and smooths the R-R time series and compares the smoothed waveform with the actual RR waveform. Thresholds are set based on the “low, medium, and high” settings that govern the degree of allowable variance between the two waveforms. Any beat that deviates significantly from the smoothed range is flagged as potential artifact.

We demonstrated that at low artifact percentages, EQ02 can be used to reliably monitor ECG and its derivatives (HR, HRV) in relatively healthy participants. This is in accordance with a previous study (Liu et al., 2013) that compared HR derived from EQ02 and Polar S810i HR monitors under 10 min each of standing, lying and sitting. However, at higher artifact percentages (50% and higher), we found discordance between the EQ02 and Holter, which did not improve after application of the automatic cleaning options provided by Vivosense. Correlations improved by selecting data with < 50% artifact or < 20% artifacts. Similarly, HRV parameters (SDNN, RMSSD, and pNN50) also showed markedly improved correlations after selection on a 3-h stretch of data with minimal artifacts. This further strengthens the finding that the quality of EQ02 is best at low artifact percentages. This agrees well with observations made by investigations into other mobile devices (Haberman et al., 2015). For example, a validation study of the Actiheart found that HR values from Actiheart were in good agreement with those of other HR monitors during rest, but errors increased during exercises of higher intensity (Haberman et al., 2015). During high intensity movements, mobile devices are more prone to artifacts, in comparison to during rest. In line, whilst investigating potential determinants of artifacts, we found that artifact percentages were lower at lower activity levels (fewer step counts), at night, in subjects with lower waist: hip ratio and also somewhat lower in females. At night, participants were lying supine and mostly asleep which might result in better contact with electrodes and/ or decreased movement. Participants with lower waist: hip ratio also had significantly lower artifact percentages possibly because of better fitting of the Equivital belt. This could also have been the case in females, since female participants wore an extra layer of underwear over the Equivital belt, which might have potentially reduced belt displacements. This suggest that the artifact content of the EQ02 were most likely attributable to motion artifacts and/or impaired skin-electrode contact.

One main limitation of our study is that it was conducted in eighteen relatively healthy participants without overt cardiac disease. A strength was that ECG was measured continuously and simultaneously using both devices over 24 h. More studies are needed to validate the EQ02 in specific groups, such as in the elderly, in large population studies and in patients with known cardiac disease. However, the susceptibility of the EQ02 to artifacts should be taken into account in such studies. Before application of Holter monitors, skin preparation is normally done with alcohol/KCl, and red dot to remove non-conductive skin layer and reduce skin impedance to minimize artifacts. Perhaps employing skin preparation techniques could also aid in minimizing artifacts with EQ02 recordings.

Summarily, we compared continuous ECG from EQ02 to the Holter over 24 h. Skin preparation, as well as clips used before application of the Holter electrodes prevents artifacts, whereas artifact management of EQ02 was done after data acquisition. We found that there was, on average, good agreement between HR and HRV values derived from EQ02 and Holter. However, its accuracy and reliability depended on the presence and quantity of artifacts. Presently, the artifact load of EQ02's ECG recordings exceeds that of the Holter. This would pose a serious limitation to its clinical use in individual patients, especially for measurements that are especially sensitive to artifacts. On the other hand, if artifacts can be properly managed, the EQ02's ability to monitor (live and/or retrospective), synchronize and store cardiac and other physiological parameters may offer potential benefits for home monitoring and/ or research purposes, as it could be useful for extensive continuous recording of ECG, HR, HRV and other physiologic data in large population studies.

## Author contributions

AA and DH conceived the study. AA, VP, DB and AM collected, analyzed, and interpreted the data. AA wrote the manuscript. All authors critically appraised the manuscript and approved its submission.

## Funding

This work was supported by the European Commission project Switchbox (grant number FP7, Health-F2-2010-259772). The funders had no role in the design of the study, collection of the data, analyses, decision to publish, or preparation of the manuscript.

### Conflict of interest statement

The authors declare that the research was conducted in the absence of any commercial or financial relationships that could be construed as a potential conflict of interest.

## Abbreviations

ANN: Average of NN intervals
ARD: Absolute relative difference
BPM: Beats per minute
ECG: Electrocardiogram
EQ02: Equivital EQ02 lifemonitor
Holter: Holter ambulatory ECG monitor
HR: Heart rate
HRV: Heart rate variability
MARD: Mean absolute relative difference
ms: Milliseconds
NN: Normal- to- normal sinus rhythm interval
NN50: Number of adjacent NN intervals with a difference less than 50 ms
pNN50: Ratio of a NN50 to total number of NN intervals
RD: Relative difference
RR: interval between the R wave peaks of the recorded QRS complex
RMSSD: Square root of the mean squared differences of successive intervals
SDNN: Standard deviation of NN intervals
SDANN: Standard deviation of 5 min averages of NN intervals
SDSD: Standard deviation of successive differences of NN intervals
SDNNi: Mean of the standard deviation of 5-min NN intervals.
