# Supplementary material for: Comparative Analysis of the Equivital EQ02 Lifemonitor with Holter Ambulatory ECG Device for Continuous Measurement of ECG, Heart Rate, and Heart Rate Variability: A Validation Study for Precision and Accuracy
Source: Front Physiol. 2016 Sep 21;7:391. doi: 10.3389/fphys.2016.00391 (PMC5030218; doi:10.3389/fphys.2016.00391)
Supplement: Supplementary file 1 [file Table1.DOCX]

**Supplementary Table 1. Per- participant characteristics of study subjects.**

| **S. No** | **Gender** | **Age** | **BMI** | **Step counts^*^** | **Waist: hip ratio** | **% Artefacts**** | **Total time in bed (minutes)** | **Sleep efficiency (%)***** |
| --- | --- | --- | --- | --- | --- | --- | --- | --- |
| 1 | M | 32 | 20.1 | 9998 | 0.91 | 41.70 | 431 | 93 |
| 2 | M | 57 | 21.45 | 10034 | 0.87 | 22.15 | 524 | 89 |
| 3 | M | 46 | 23.85 | 7915 | 0.88 | 1.93 | 413 | 97 |
| 4 | F | 19 | 20.2 | 7161 | 0.87 | 56.45 | 518 | 89 |
| 5 | M | 20 | 23.05 | 5017 | 0.73 | 34.08 | 680 | 99 |
| 6 | M | 26 | 21.22 | 11496 | 0.80 | 15.68 | 512 | 96 |
| 7 | F | 24 | 22.51 | 14265 | 0.82 | 25.87 | 505 | 94 |
| 8 | F | 24 | 19.80 | 5423 | 0.77 | 13.36 | 513 | 100 |
| 9 | F | 22 | 22.96 | 13513 | 0.76 | 5.81 | 477 | 98 |
| 10 | M | 25 | 20.78 | 5371 | 0.83 | 7.60 | 698 | 98 |
| 11 | F | 27 | 22.56 | 10691 | 0.75 | 5.23 | 500 | 96 |
| 12 | F | 23 | 23.92 | 10771 | 0.74 | 7.27 | 507 | 93 |
| 13 | M | 24 | 24.38 | 12890 | 0.80 | 18.10 | 556 | 93 |
| 14 | F | 29 | 21.73 | 6183 | 0.71 | 4.00 | 650 | 91 |
| 15 | F | 21 | 19.62 | 12521 | 0.74 | 8.94 | 953 | 99 |
| 16 | M | 25 | 30.34 | 9580 | 0.92 | 24.85 | 961 | 100 |
| 17 | M | 29 | 23.89 | 8908 | 0.91 | 30.91 | 514 | 97 |
| 18 | M | 24 | 22.03 | 11907 | 0.81 | 18.12 | 532 | 86 |

M=males, F= females

*total number of steps taken in 24 hours

** percentage artefacts present in the raw data, excluding charging times

*******Sleep efficiency as calculated by Fitbit program as a composite of time the participant went to bed, time to fall asleep, number of awakenings and restless periods during sleep, the total time in bed and the actual sleep time**.**
