# Supplementary figures and images for: Comparative Analysis of the Equivital EQ02 Lifemonitor with Holter Ambulatory ECG Device for Continuous Measurement of ECG, Heart Rate, and Heart Rate Variability: A Validation Study for Precision and Accuracy
Source: Front Physiol. 2016 Sep 21;7:391. doi: 10.3389/fphys.2016.00391 (PMC5030218; doi:10.3389/fphys.2016.00391)

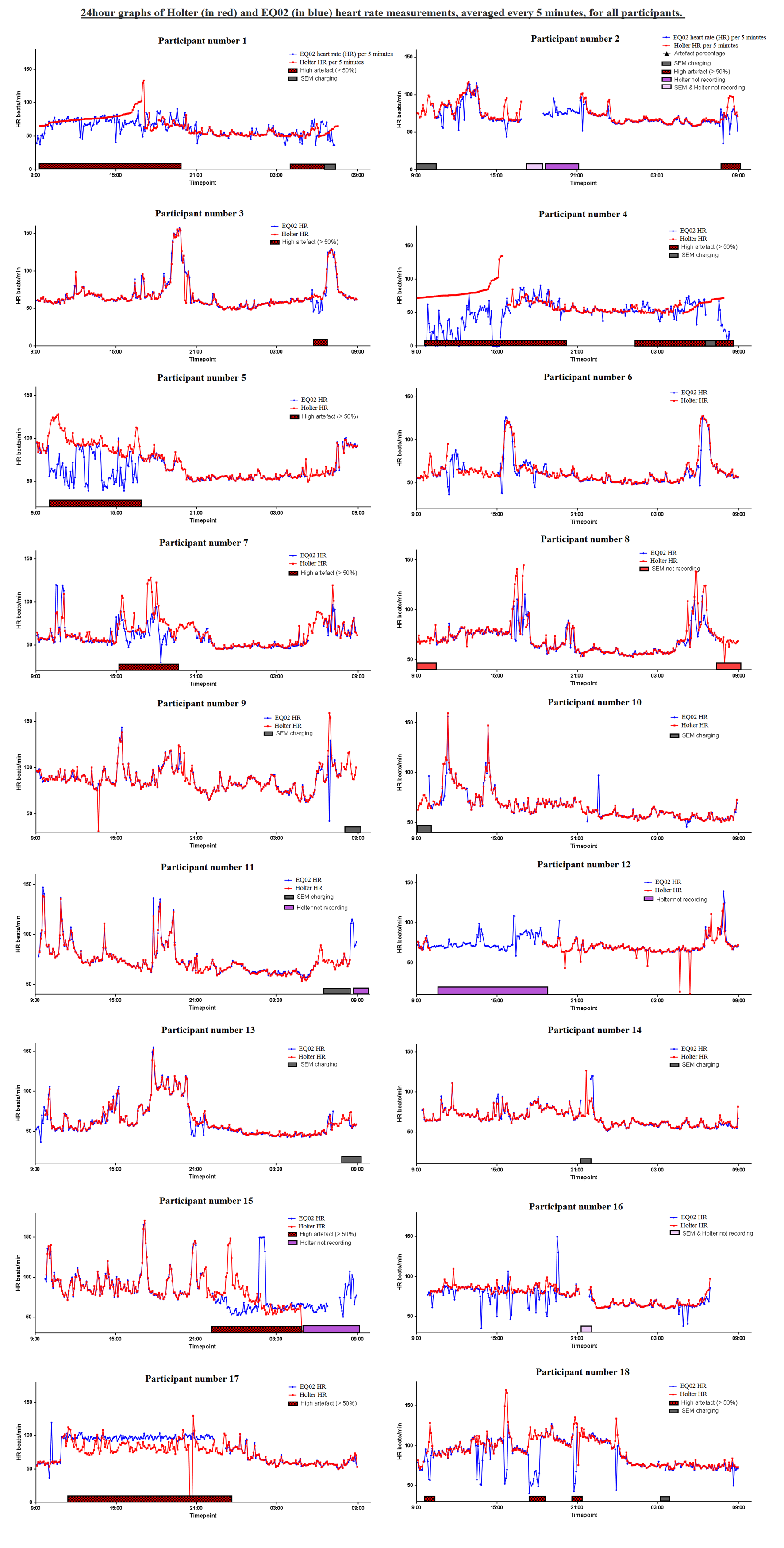

Supplement: Supplementary file 2 [file Image1.TIF]
